# 40 years publishing on women’s health and the *Birth in Brazil* survey

**DOI:** 10.1590/0102-311XEN044624

**Published:** 2024-04-29

**Authors:** Maria do Carmo Leal

**Affiliations:** 1 Escola Nacional de Saúde Pública Sergio Arouca, Fundação Oswaldo Cruz, Rio de Janeiro, Brasil.

This year, we celebrate the 40th anniversary of CSP and are pleased to find that over this period women’s health represented more than 10% of all its 8,255 publications (articles, editorials, book reviews, brief communications, and others, according to the database SciELO; https://search.scielo.org/). Publications featured an enormous diversity of themes, such as gender and race-related violence and inequities; periodic examinations of health including breast and cervical cancer, and other pathologies such as diabetes, hypertension, and obesity; as well as several other topics, such as the human rights agenda and the analysis of policies and programs addressing women’s health.

The most prominent issue within the range of topics addressing women’s health was the sub-theme of sexual and reproductive health, with 86% of all the published knowledge produced falling within this category.

Recognition of the importance of women’s health (and especially of their sexual and reproductive health) as fundamental public health themes is consistent with the magnitude of this field within the Brazilian Unified National Health System (SUS). In the hospitalization statistics of the Brazilian Hospital Information System, chapters XV and XVI - “Pregnancy, Childbirth, Puerperium”, and “Some Conditions Originating in the Perinatal” period, respectively - corresponded to 20.1% of all hospitalizations in Brazil in 2023-2024 ^1^. No less relevant is the expression of this area in primary care. Data published in the Brazilian National Program for Improvement of Access and Quality of Basic Care (PMAQ-AB, acronym in Portuguese) in 2013 show that 28,056 of the 30,523 teams (92%) that participated in the study actively worked in women’s and children’s health care ^2^.

CSP’s alignment with the aspects related to caring for women’s sexual and reproductive health shows that this journal is aware of the health needs of the Brazilian population, reflecting the demands of this area in its scientific focus. The main topics addressed by the publications on sexual and reproductive health included women’s and children’s health, pregnancy, and childbirth (60% of the total), followed by infant mortality, abortion, contraception, among others. Notably, the last 10 years have seen a reduction in the number of articles on infant mortality and contraception, whereas the percentages of publications on childbirth and severe maternal morbidity have increased.

The excellent work by the editors of CSP and its body of reviewers led us to choose this scientific journal to disseminate the results of the *Birth in Brazil: National Survey into Labor and Birth* (*Birth in Brazil I*). The *Birth in Brazil I* survey indisputably configured a milestone in the Brazilian scientific production on sexual and reproductive health. The first results of the survey were published in a thematic issue, the main article of which, *Obstetric Interventions During Labor and Childbirth in Brazilian Low-risk Women* ^3^ has almost 61,000 downloads and 550 citations, being one of the most cited articles by readers of the journal according to data from Google Scholar (https://scholar.google.com.br/scholar?cites=11612483559201017160&as_sdt=2005&sciodt=0,5&hl=en).

For the first time, *Birth in Brazil I* showed the panorama of labor and birth care, the exorbitant rates of cesarean sections, the precariousness of perinatal indicators for the public and private healthcare sectors, and the obstetric violence women and their babies undergo due to their subjection to painful and unnecessary interventions and lack of proper care. Most of these practices were at odds with the scientific evidence and recommendations from the World Health Organization (WHO). Awareness of this situation was fundamental for Brazil to mobilize and seek mechanisms for improving it. The public sector created the Stork Network program ^4^, program with the following guidelines: to welcome, assess, and classify the risk and vulnerability of women upon arrival at a maternity hospital; to link pregnant women during prenatal care to referral units for childbirth and ensure their safe transportation; to offer good practices and safety in labor and birth care; and to provide children aged 0 to 24 months with good-quality, problem-solving health care. Regarding the private sector, the Brazilian National Supplementary Regulatory for Private Health Insurance and Plans (ANS, acronym in Portuguese), in conjunction with the Hospital Israelita Albert Einstein and the Institute for Healthcare Improvement (United States), created the Adequate Childbirth program ^5^, which aimed to support, instrumentalize, and implement actions based on scientific evidence for the supplementary health sector to safely reduce the percentage of unnecessary cesarean sections and increase the quality and safety of care provide during labor and birth.

In the following years, our research group had the opportunity to evaluate these programs and assess some of the advances they had achieved in reducing cesarean sections, increasing access to good practices, and decreasing non-recommended interventions in childbirth care in the private hospitals that were assessed. It also found a reduction in geographical, social, and racial inequities regarding access to appropriate technologies for labor and birth care in the public sector ^6^ ^,^ ^7^.

If the diagnosis brought forth by the *Birth in Brazil I* survey had provided important guidelines for public policies at the Brazilian Ministry of Health and the supplementary health sector for women’s and children’s health, its continuity was essential to monitoring the results achieved and in maintaining assessment of these indicators.

In view of these achievements, the Brazilian Ministry of Health proposed a second national survey along the same lines as the first, called *Birth in Brazil II: National Research on Abortion, Labor and Childbirth* (*Birth in Brazil II*), for 2021-2023. Considering the high proportion of unwanted pregnancies in the *Birth in Brasil I* survey and the maternal morbidity and mortality rates associated with abortions in Brazil, this new study proposed to inclusion of women admitted to hospitals for abortions.

Understanding gained during this period led us to nest several studies in *Birth in Brazil II* in order to obtain a significant number of cases that would enable a more in-depth analysis of maternal death near mis*s* and maternal death and a qualitative analysis of the casas of abortion cases studies. In this issue of CSP, we present the Thematic Section with articles on the *Birth in Brazil II* survey protocol and the studies integrated with it.

The path of CSP and the scientific quality of its publications make this journal a heritage of Brazilian science in public health. We wish the CSP many more years of work. We would like to express our warmest appreciation for science, SUS and CSP!
